# Mature Cystic Teratoma in a Newborn With Down Syndrome

**DOI:** 10.7759/cureus.63878

**Published:** 2024-07-05

**Authors:** Kavimozhy Ilakkiya Poyyamozhy, Srinivas Srirampur, Pranay Kumbha, Nagarjuna Kumbha

**Affiliations:** 1 Pediatric Surgery, Gandhi Medical College and Hospital, Hyderabad, IND; 2 Medicine, Coimbatore Medical College and Hospital, Coimbatore, IND

**Keywords:** case report, abdominal cystic lesion, renal hilum, retroperitoneal teratoma, down syndrome neonate

## Abstract

Retroperitoneal teratomas are uncommon neoplasms that cause diagnostic dilemmas in neonates during the evaluation of abdominal cystic mass lesions. Down syndrome (DS) is a chromosomal abnormality with an extra third copy of chromosome 21. Due to chromosomal instability, DS is inferred to be a cancer predisposition syndrome. The malignancy pattern in DS is unique with higher incidence of hematological malignancies and solid tumors are rarely reported. Down syndrome neonate was incidentally diagnosed with a retroperitoneal cystic lesion along the left kidney during evaluation for poor feeding on ultrasonography, raising suspicion of an adrenal hemorrhagic cyst. CECT abdomen and pelvis revealed complex cystic lesions along the left renal hilum, with the possibility of cystic neuroblastoma, retroperitoneal cystic teratoma, and cystic lymphangioma. Tumor markers were within normal limits. Surgical exploration revealed a left renal hilar solitary cystic mass lesion, which was excised in toto with a probable intraoperative diagnosis of cystic lymphangioma. The postoperative course was uneventful. Histopathological examination confirmed that the lesion was a mature cystic teratoma. The child is doing well postoperatively at the one-year follow-up. Neonatal retroperitoneal teratomas are unusual neoplasms. A favorable outcome can be achieved, with early diagnosis and treatment.

## Introduction

Primary retroperitoneal teratomas are rare tumors. According to Sarangi et al., they are very uncommon in neonates [1]. Augé et al. found that most of these tumors are benign, but they can be malignant, especially in newborns compared to older children [2]. These tumors can be challenging to diagnose when they are large and distort the local anatomy. In such cases, a definitive diagnosis may not be possible before surgery, leading to diagnostic difficulties when evaluating large abdominal cystic masses in children.

Neonates with Down syndrome (DS) exhibit a distinct neoplastic profile, characterized by a higher occurrence of retroperitoneal teratomas and a lower occurrence of sacrococcygeal teratomas compared to the general population, as evidenced by Kobayashi et al. [3]. Despite the rarity of retroperitoneal teratomas in neonates, it is crucial to acknowledge the unique pattern of neoplasms observed in individuals with Down syndrome.

## Case presentation

**Figure 1:**
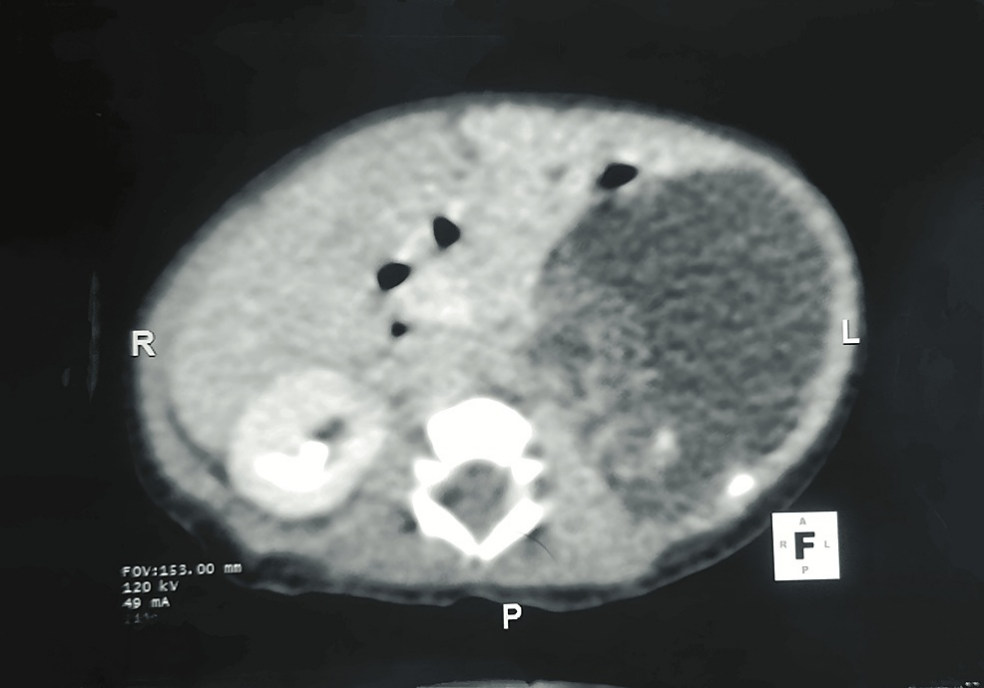
Contrast-enhanced computed tomography of abdomen depicting a huge retroperitoneal cystic mass lesion. A large cystic mass lesion was noted in the retroperitoneum on left side in suprarenal location with few calcifications and a solid component within the cystic lesion.

A six-day-old female child with Down syndrome was referred to a pediatric surgical unit for an incidentally diagnosed retroperitoneal cystic lesion. This was a term child with a birth weight of 2.1 kg, the first child by birth order, and delivered by spontaneous vaginal delivery. The mother has lost prenatal visits since the seventh month of pregnancy, and no sonography reports are available. Following delivery, since day four of life, the child has been admitted to the NICU for feeding intolerance and poor activity. On examination, the child typically had Down syndrome features, with a palpable, vague mass on the left side of the upper abdomen. A 7×4.1 cm retroperitoneal cystic lesion with septations and peripherally solid components was revealed by ultrasound. The lesion was located inferior to the stomach, anterior to the left kidney, and posterior to the pancreatic tail. The adrenal gland could not be separately visualized from the lesion. Because the characteristics indicated an adrenal hemorrhagic cyst, the child was assessed with serial USG at regular intervals while being closely observed. There was no remarkable change compared with previous findings. Abdominal computed tomography (CT) showed a large, well-defined, hypodense, poorly enhanced cystic mass lesion occupying the retroperitoneum on the left side in a suprarenal location (Figure 1).

**Figure 2:**
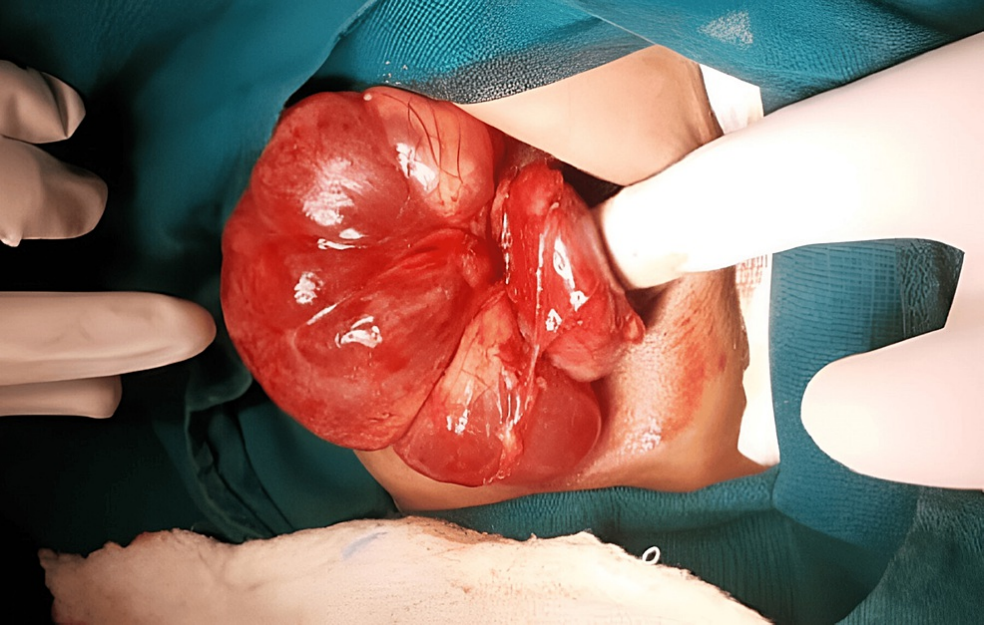
Intraoperative image revealed the cystic lesion in the left renal hilar region. Solitary well-defined cystic mass of 12x6x7 cm in the left renal hilar region with surgeon finger pointing the left kidney.

It was noted to abut and displace the left adrenal gland superiorly, the left kidney, and the left renal vein inferiorly, almost abutting the abdominal aorta medially and causing displacement of the body and tail of the pancreas. The narrow septations and sparse calcifications observed within suggest cystic neuroblastoma, cystic teratoma, and cystic lymphangioma as possible differential diagnoses. The levels of human chorionic gonadotropin and alpha-fetoprotein were within normal ranges. A solitary, well-defined cystic mass measuring 12×6×7 cm was found during surgical exploration in the left renal hilar region (Figure 2).

**Figure 3:**
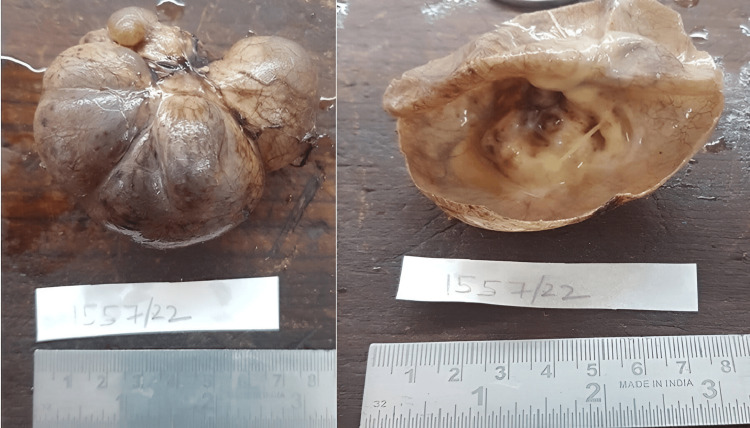
Macroscopic image of the specimen. The external surface was grey-white to grey-brown, bosselated and congested, and dilated blood vessels were seen. The cut surface revealed a unilocular cyst, with a white-to-yellow solid component observed in one focal area.

There was no sign of infiltration or vascular encasing. The left kidney, adrenal gland, and pancreatic tail were normal. It was challenging to dissect renal hilum vessels, and with meticulous dissection, the mass was excised in toto (Figure 3).

**Figure 4:**
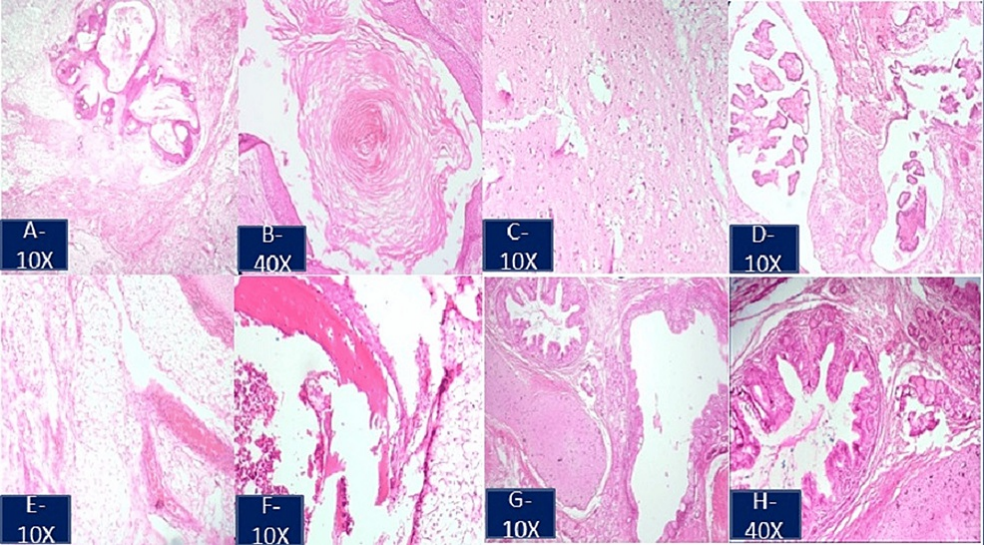
Histopathological image of the excised specimen stained with hematoxylin and eosin depicts the derivatives of three germ layers. (A-D) Ectodermal components, stratified squamous epithelium, adnexal structures, keratin material, neuroglial tissue, and choroid plexus are seen. (E-G) Mesodermal components, sheets of mature adipocytes, blood vessels, muscle bundles, osteoid, marrow elements, and cartilage are seen. (G, H) Endodermal components, intestinal-type glands seen as derivatives of three germ layers.

Its cystic nature with thin septa within it raised the possibility of cystic lymphangioma. The postoperative course was uneventful. A cut section of the specimen revealed an unilocular cyst with solid components. Histopathological examination confirmed that the lesion was a mature cystic teratoma (Figures 4A-4H). The child is doing well postoperatively during the one-year follow-up.

## Discussion

Among germ cell tumors, retroperitoneal teratoma accounts for 3.5-4% of all cases in children with female preponderance. They are usually secondary neoplasms and primary retroperitoneal neoplasms are extremely rare estimated to be 1-11% [1]. Neonatal retroperitoneal tumors have a higher proportion of malignant teratomas than other locations, such as the sacrum and coccyx, heart, neck, mediastinum, and abdomen. Most of these tumors are located in the pararenal area and tend to be more prevalent on the left side. These tumors display symptoms relevant to the location of origin, or they may remain asymptomatic until they become very large. The tumor may present late with infection and rupture, leading to complications such as abscess formation and peritonitis, which may be life-threatening. In neonates, Augé et al. reported that the proportion of malignant teratomas is greater for retroperitoneal tumors than other sites, including the sacrum and coccyx, heart, neck, mediastinum, and abdomen [2]. In neonates, most teratomas occur mainly in the sacrococcygeal area, followed by the anterior mediastinum. After reviewing the literature, Kobayashi et al. reported that neonates with Down syndrome (DS) have a unique neoplastic profile with a greater frequency of retroperitoneal teratomas and a lower incidence of sacrococcygeal teratomas than the general population [3].

Prenatal ultrasonography may be highly helpful for early diagnosis. Prenatal assessment aids in postnatal strategic management and effective counseling for prospective parents. This promotes early, prompt intervention and favorable outcomes [4]. Surgery is the mainstay of treatment in cases of teratoma, irrespective of the nature, extent, or even when involving vital structures. Retroperitoneal neuroblastoma may also manifest as a cystic tumor prenatally. Nuchtern et al.'s study demonstrates that the strategy of surgical exploration has been replaced by expectant observation of infants aged six months with small cystic tumors and in low-grade neuroblastoma, as tumor regression has been detected in most patients [5]. In our case, failed prenatal visits and fetal sonography were not available. The initial imaging method used to assess any pediatric cystic abdominal lesion is often abdominal ultrasound. An adrenal hemorrhagic cyst, which was our first diagnosis, was disregarded because further USGs did not reveal any changes in the lesion's size or form. Computed tomography (CT) of the abdomen and pelvis suggested the likelihood of cystic neuroblastoma, cystic teratoma, and cystic lymphangioma. A precise diagnosis is not always feasible before surgery. Preoperative assessment with CT and/or MRI is crucial in determining the extent and relationship of the tumor with the great vessels. Rattan et al. highlight that the CT scan may exaggerate the tumor’s level of adhesion to nearby structures compared with that observed during the preoperative examination [6]. In light of this, the results of the CT scan should not be used to forbid surgical exploration of the tumor. In cystic neoplasms, surgical excision remains the treatment of choice, irrespective of location.

In this case, the lesion was located in close proximity to the renal hilar vasculature. The tumor was resected, sparing the renal vessels. Adequate resection is possible and necessary for the cure. Yang et al. described complete resection as not always possible when they are in intricate relation to vital structures that can add to surgical misadventures [7]. They reported a high perioperative complication rate in retroperitoneal teratoma resection. Our neonate underwent complete excision of the tumor with no perioperative or postoperative complications. The preoperative finding of a multilocular septate cystic lesion affected our decision to diagnose the patient with cystic lymphangioma. Only histology could identify the tumor as a mature cystic teratoma. The histopathology is of utmost importance in teratomas because evidence of immature and malignant features determines the need for adjuvant therapies and vigilant follow-up for recurrence, although recurrence is scarcely seen in mature teratomas. Tumor markers were within normal limits in our patient. The mature teratoma does not cause an elevation of tumor markers, whereas immature teratoma could be associated with elevated alpha-fetoprotein (AFP), especially if it contains microscopic foci of the yolk sac tumor. The prognosis is good after complete surgical excision of a mature cystic teratoma.

## Conclusions

Renal hilar mature cystic teratomas are rare in neonates and unfamiliar locations. Despite being unusual, retroperitoneal teratomas should be considered as a differential diagnosis of an abdominal cystic mass in a newborn. It is also important to understand the distinct neoplasmic profile observed in Down syndrome as it differs widely from a normal neonate. Keeping this in mind will be quite valuable when evaluating the patient. Prompt surgical management will have a good outcome.

## References

[1] Sarangi PK, Hui P, Mangaraj PD, Kumar S (2017). Retroperitoneal teratoma in infancy: report of an unusual entity. J Med Diagnostic Meth.

[2] Augé B, Satgé D, Sauvage P, Lutz P, Chenard MP, Levy JM (1993). Retroperitoneal teratomas in the perinatal period. Review of the literature concerning a neonatal, immature, aggressive teratoma. [Article in French]. Ann Pediatr (Paris).

[3] Kobayashi T, Sakemi Y, Yamashita H (2014). Increased incidence of retroperitoneal teratomas and decreased incidence of sacrococcygeal teratomas in infants with Down syndrome. Pediatr Blood Cancer.

[4] Asai S, Ishimoto H, Kim SH (2009). Prenatal diagnosis of retroperitoneal teratoma: a case report and review of the literature. Fetal Diagn Ther.

[5] Nuchtern JG, London WB, Barnewolt CE (2012). A prospective study of expectant observation as primary therapy for neuroblastoma in young infants: a Children's Oncology Group study. Ann Surg.

[6] Rattan KN, Kadian YS, Nair VJ, Kaushal V, Duhan N, Aggarwal S (2010). Primary retroperitoneal teratomas in children: a single institution experience. Afr J Paediatr Surg.

[7] Yang T, Li H, Li J (2019). Surgical risk factors of retroperitoneal teratoma resection in children. J Pediatr Surg.

